# Knowledge and Practice of Scar Treatment Among Health Care Physicians in Saudi Arabia

**DOI:** 10.7759/cureus.60057

**Published:** 2024-05-10

**Authors:** Ziyad Alharbi, Ranad M Khashab, Eyas Farran, Maha S Bamatraf, Maan T Almaghrabi, Sherif F Khamis, Kausar D Ahmed

**Affiliations:** 1 Clinical Sciences, Fakeeh College for Medical Sciences, Jeddah, SAU; 2 Plastic Surgery and Burn Unit, Dr. Soliman Fakeeh Hospital, Jeddah, SAU; 3 Medicine, Fakeeh College for Medical Sciences, Jeddah, SAU; 4 Pediatric Plastic Surgery and Burns, King Abdulaziz Medical City, Riyadh, SAU

**Keywords:** scar treatment, scar prevention, scar evaluation, hypertrophic scars, keloid scars

## Abstract

Background

The processes of wound healing and scar formation are complex phenomena that are determined by an intricate interplay of molecules and cells. A deviation from the anticipated trajectory of scarring can lead to the formation of hypertrophic scars and keloids. A wide range of therapeutic methodologies have been employed in the treatment of scars. This research paper seeks to enhance patient outcomes and the efficacy of scar repair as a whole by determining the knowledge of scar treatment and implementation in clinical practice in Saudi Arabia and thereby incorporating scientific findings into practical settings.

Materials and methods

This cross-sectional study, which included 237 participants, aimed to provide descriptive data on the knowledge and common practice of Saudi Arabian healthcare physicians with regard to scar prevention, treatment, and evaluation during the period from November 15, 2023, to December 11, 2023.

Results

In routine clinical practice, the most commonly employed subjective method for scar assessment is patient and observer scar assessment (162 (68.4%)) while the Modified Vancouver Scar Scale (91 (38.4%)) was commonly used for research purposes. However two-dimensional photography is the most frequently employed objective method in clinical practice (54 (22.8%)) and biomechanical properties (58 (24.5%)) for research purposes. Silicone scar therapy in the form of sheets or gel is the primary preventive measure in the prevention of keloids/hypertrophic scars across various patient populations. Corticosteroid injections and silicone are primary interventions within the initial 18-month period.

Conclusion

Although significant progress has been made in the field of scar management, standardization of procedures and increased adherence to evidence-based guidelines are still required.

## Introduction

In the realm of plastic surgery, scarring is a prevalent and consequential issue, frequently stemming from burns, traumatic incidents, and surgical interventions. Scars can induce significant psychological distress, functional impairment, pain, and aesthetic dissatisfaction, in addition to their obvious physical consequence [1]. Wound healing and scar formation are phenomena that are influenced by an intricate interplay of cellular and molecular processes. The process undergoes discrete stages, which comprise hemostasis, inflammation, proliferation, and remodeling [2]. Scarring that deviates from the expected course may result in the development of hypertrophic scars and keloids. It is critical to understand the pathophysiology underlying scars to implement efficient scar management strategies to reduce these negative consequences and enhance patient results [3]. A diverse array of therapeutic approaches has been utilized for the management of scars. Silicone gel sheeting, surgical excision, pressure therapy, and laser therapy are among the numerous options that are accessible [4,5]. Yet, ascertaining the most effective course of treatment continues to be a formidable task, given that every approach possesses distinct merits, drawbacks, and potential hazards. The effectiveness of these treatments may differ based on patient characteristics, scar type, and location [6].

Atrophic scars, like hypertrophic scars and keloids, pose a distinct array of difficulties. An exploration of platelet-rich plasma (PRP), microneedling, subcision, and laser resurfacing has been undertaken in an effort to enhance the visual appeal and tactile qualities of atrophic scars [7]. PRP has demonstrated effectiveness in various areas, mostly due to its capacity to produce high levels of growth factors and stimulate cell proliferation and angiogenesis, hence facilitating tissue regeneration and limiting the formation of scars [8]. Research has shown that it can effectively treat a number of problems, including alopecia, acne, melasma, burn scars, and skin hyperpigmentation [9]. Many over-the-counter and prescription topical treatments are available that promise to reduce discomfort, enhance the look of scars, and speed the healing process of wounds. The benefits of topical medicines include improved adherence, localized product administration, and a decreased impact from first-pass metabolism [10]. In that regard, a scar gel containing the pharmacodynamically active substances Allium cepae, allantoin, and heparin (Contractubex, Merz Pharma GmbH & Co., Frankfurt, Germany) has shown promise in treating unattractive and elevated scars and is used as a preventative measure against scar formation [11]. Nonetheless, the absence of standardized instruments for scar evaluation results in inconsistent assessments of scar attributes including color, pliability, thickness, and irregularity. It is essential to develop dependable and unbiased measuring instruments to accurately assess scars and monitor the efficacy of treatments [12]. Although scar management has made significant strides, corrective surgery continues to be the predominant intervention in certain instances. However, difficulties persist despite surgical interventions, especially with pathological scars such as keloids, which are prone to recurrence [13].

In light of the intricate nature and formidable obstacles entailed in scar management, it is critical to evaluate the expertise and application of medical practitioners in this domain. Gaining insight into healthcare professionals' present practices, treatment inclinations, and possible knowledge deficiencies can facilitate the identification of areas requiring enhancement and provide direction for the creation of evidence-based guidelines and educational initiatives [13]. The objective of this research paper is to investigate the understanding and application of scar treatment by healthcare physicians, in an effort to improve patient outcomes and the overall effectiveness of scar management.

## Materials and methods

Design

This cross-sectional study, conducted at Dr. Soliman Fakeeh Hospital, Jeddah, Saudi Arabia, sets out to provide descriptive data regarding the knowledge and common practice of healthcare physicians in Saudi Arabia regarding the evaluation, prevention, and treatment of scars. Data collection was carried out through November 15, 2023, and ended on December 11, 2023. A total sample of 237 participants of healthcare physicians were surveyed using a self-administered electronic questionnaire that was adapted from a previous study done in Graz, Austria [14]. Upon obtaining permission for the use of the questionnaire from the authors face validation was carried out. Disclosure of responses will be given in detail based on “Eysenbach’s Checklist for Reporting Results of Internet E-Surveys (CHERRIES)” [15]. The checklist is shown in Table 3 in the Appendix. Following clearance by the Dr. Soliman Fakeeh Hospital Institutional Review Board (approval no. 512/2023), all participants' verbal and written agreement was taken following an explanation of the study's duration, purpose, and content.

Data questionnaire

The questionnaire contains three main sections, the first part contains consent to participate in the study; the second part requires filling out personal information such as specialization, gender, age, etc.; the third part contains eight questions assessing the knowledge of health care physicians regarding scars seen in their practice and how they evaluate, manage, and treat it. The questions were divided into four further sections. First, methods of scar evaluation in daily clinical practice or for research purposes, either by subjective methods or by objective tools. Second, scar prevention has three presentations: expected uneventful wound healing, increased risk of developing hypertrophic scars, and a previous history of keloid disease. Third, questions regarding the treatment of hypertrophic scars less than 18 months after the initial presentation and more than 18 months after the initial presentation. Last is the treatment of keloids. The participants were asked whether or not they operate. If they responded yes, they were then directed to questions discussing their treatment options before and after surgery. If the participants did not opt for surgery, they were asked about their non-surgical treatment options. No randomization of items was done and a convenience sample was taken. All healthcare physicians from both genders and all nationalities currently practicing in KSA were included, and all others were excluded. The available answer options were adapted from the original study, which was based on the available methodology and recommendations reported in the current literature. The questionnaire was adapted to an online administration database system (Google Forms). Each question had limited predetermined responses. Multiple selections were allowed and were marked as required. For every item, there was a "other" option in case none of the response choices applied. The same person could not submit more than one entry, and each link could only be used once (there was no way to use the same link again after the survey was finished). Questionnaires that were not completed were excluded from the study. There were no incentives for survey participation.

Data analysis

Once data collection was complete it was transferred to a Microsoft Excel file with representative figures generated. The resultant data was converted to numbers in frequencies and percentages for ease of readability and frequency distribution of selections and then analyzed through IBM SPSS Statistics for Windows, Version 24 (Released 2016; IBM Corp., Armonk, New York, United States). P-values < 0.05 were considered statistically significant.

## Results

A summary of the study's sample demography is represented in Table 1. The sample consists of 237 healthcare physician participants; with respect to gender, 125 (52.7%) participants identify as female, whereas 112 (47.3%) are male. In relation to age, the data reveals that 88 (37.1%) individuals belonged to the age group of 25 to 35 years, 54 individuals (22.8%) were between the ages of 35 and 45 years, and the smallest proportion of 17 individuals (7.2%) were in the age group of 45 to 50 years. In terms of professional experience, the study reveals that 86 (36.3%) participants hold the position of consultant, 64 (27%) are resident physicians, 35 (14.8%) are specialists, 31 (13.1%) are medical interns, and a mere 21 (8.9%) are general practitioners. Concerning region of practice, the results indicate that the western region is inhabited by the majority of 144 participants (60.8%), followed by the central region with 32 individuals (13.5%), the southern region with 29 individuals (12.2%), the northern region with 22 individuals (9.3%), and the eastern region with 10 individuals (4.2%). With respect to healthcare environments, the data reveals that the majority of participants, 138 (58.2%), are employed in public hospitals, while 39 (16.5%) are affiliated with private hospitals, and 36 (15.2%) are affiliated with primary health care centers.

**Table 1:** Demographic characteristics of participants N: number of individuals; %: percentage

Demographics	N (%)
Gender	
Female	125 (52.7%)
Male	112 (47.3%)
Age group	
18-25	33 (13.9%)
25-35	88 (37.1%)
35-45	54 (22.8%)
45-50	17 (7.2%)
50+	45 (19.0%)
Level of expertise	
Consultant	86 (36.3%)
Resident	64 (27.0%)
Specialist	35 (14.8%)
Medical interns	31 (13.1%)
General practitioner	21 (8.9%)
Region of practice	
Western Region	144 (60.8%)
Central Region	32 (13.5%)
Southern Region	29 (12.2%)
Northern Region	22 (9.3%)
Eastern Region	10 (4.2%)
Health care setting	
Public hospitals	138 (58.2%)
Primary health care centers	36 (15.2%)
Private hospitals	39 (16.5%)
Private clinics	21 (8.9%)
Others	3 (1.3%)
Total	237 (100%)

The specialties of the physicians who participated are shown in Table 2. Dermatology was the most prevalent among the participants, as indicated by 57 individuals (24.1%) identifying it as their medical specialty. Family medicine was the second most common, with 31 (13.1%), followed by plastic surgery with 29 individuals (12.2%). Orthopedics and emergency medicine were the least frequent specialties, with 6 (2.5%) and 3 (1.3%), respectively.

**Table 2:** Medical specialties

Variable	N (%)
Plastic surgery	29 (12.2%)
Dermatology	57 (24.1%)
General surgery	22 (9.3%)
Emergency medicine	3 (1.3%)
Ear nose throat (ENT)	10 (4.2%)
Family medicine	31 (13.1%)
Internal medicine	23 (7.2%)
Orthopedic surgery	6 (19.0%)
Pediatrics	18 (7.6%)
Obstetrics	16 (6.8%)
Other	20 (8.4%)

Methods of scar evaluation

Subjective Evaluation Score

**Figure 1:**
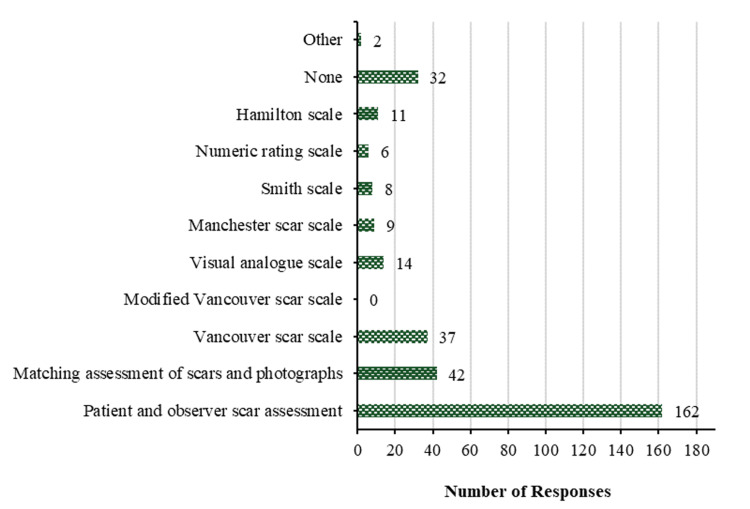
Subjective methods in daily clinical practice

In routine clinical practice, the most commonly employed subjective method for scar assessment is patient and observer scar assessment by 162 (68.4%), closely trailed by matching assessment of scars and photographs by 42 (17.7%). The Vancouver Scar Scale is utilized by 37 (15.6%), the visual analog scale by 14 (5.9%), the Hamilton scale by 11 (4.6%), the Manchester scar scale by 9 (3.8%), the Smith scale by 8 (3.4%), and numeric rating scale by 6 (2.5%). However, 32 (13.5%) mentioned using none of the mentioned scales, and 2 (0.8%) specified using different approaches. A summary of all responses is shown in Figure 1.

**Figure 2:**
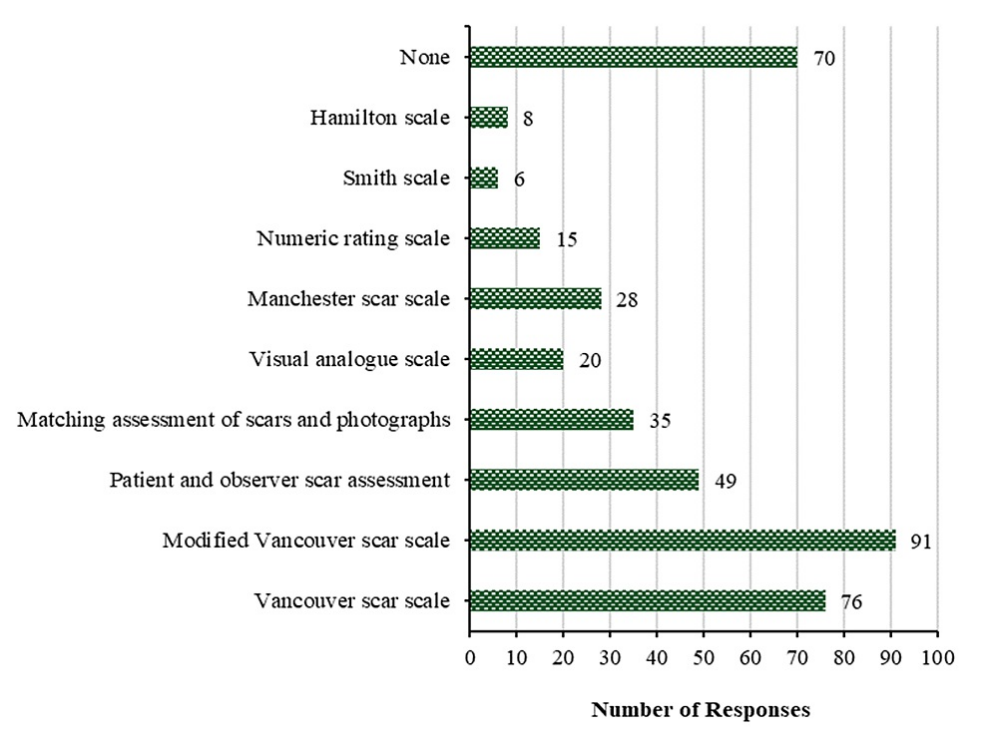
Subjective methods for research purposes

Figure 2 shows physicians' preferred subjective methods for research purposes, the predominant approach is the Modified Vancouver Scar Scale (91 (38.4%)), followed by the Vancouver scar scale (76 (32.1%)). While the least frequently used methods for research purposes are the visual analog scale, numeric rating scale, Hamilton scale, and Smith scale, with percentages of 20 (8.4%), 15 (6.3%), 8 (3.4%), and 6 (2.5%), respectively. None of the mentioned methods is opted for by 70 (29.5%) for research purposes.

Objective Evaluation Score

**Figure 3:**
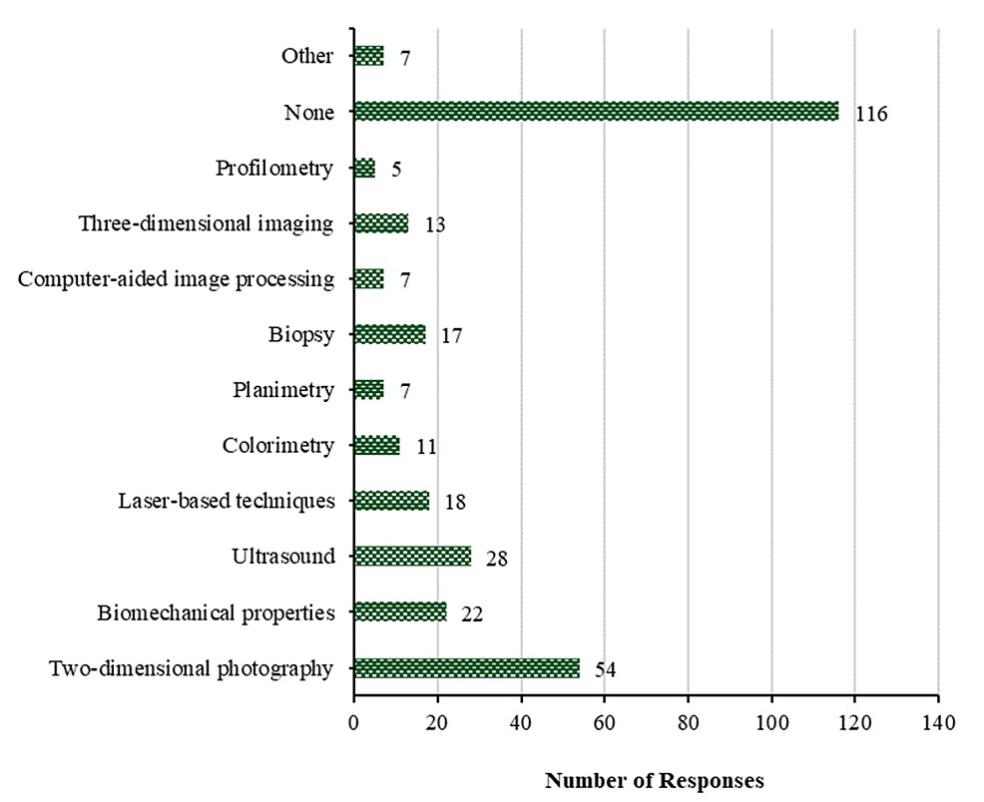
Objective methods in daily clinical practice

With respect to objective methods utilized in routine clinical practice to assess scars, the data indicates that two-dimensional photography is the most frequently employed method (54 (22.8%)), followed by ultrasound (28 (11.8%)), biomechanical properties (22 (9.3%)), laser-based techniques (18 (7.6%)), and biopsy (17 (7.2%)). The least frequently utilized objective methods in daily clinical practice are three-dimensional imaging and colorimetry with 13 (5.5%) and 11 (4.6%), respectively, and each of planimetry, computer-aided image processing, and other methods with 7 (3.0%), and profilometry with only 5 (2.1%). Conversely, 116 individuals (48.9%) do not employ any objective method in their routine clinical practice when assessing scars. Figure 3 shows the chosen objective methods in daily clinical practice.

**Figure 4:**
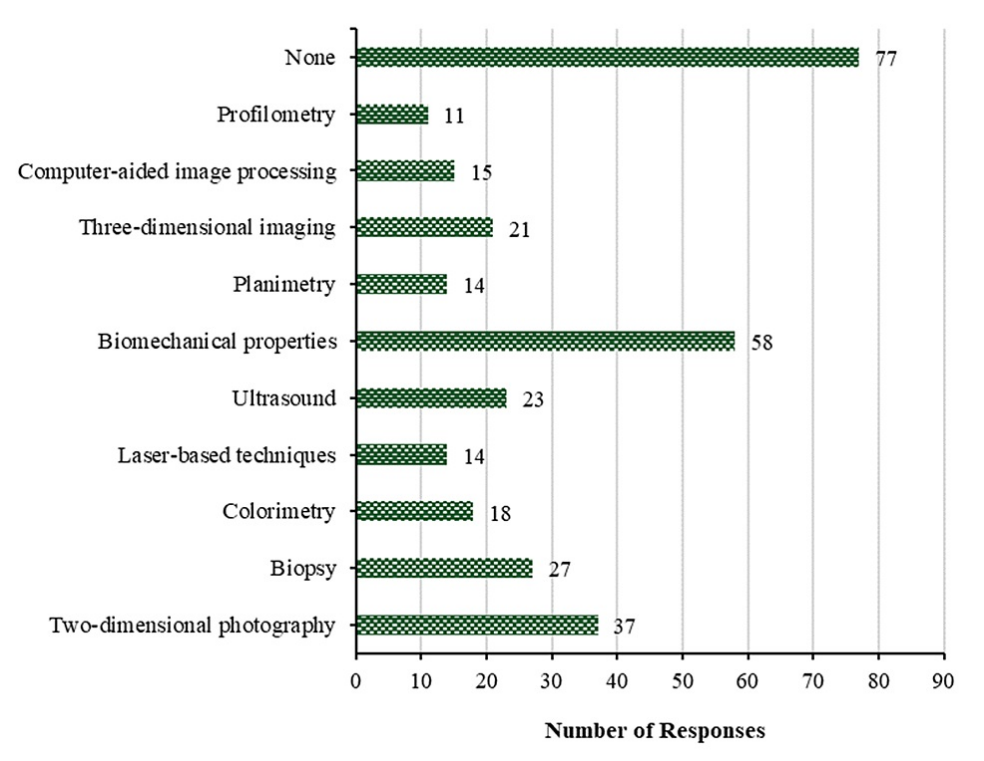
Objective methods for research purposes

In relation to the assessment of scars for research objectives, the data reveals that biomechanical properties are the most commonly used objective method with 58 (24.5%). This is followed by two-dimensional photography with 37 (15.6%), biopsy with 27 (11.4%), ultrasound with 23 (9.7%), and three-dimensional imaging with 21 (8.9%). On the contrary, in the realm of scientific research, colorimetry and computer-aided design are the least frequently employed objective techniques for assessing scarring with 18 (7.6%) and 15 (6.3%), respectively. In contrast, 77 participants (32.5%) do not utilize any objective method to evaluate scars. Figure 4 demonstrates the physicians' responses regarding objective methods for research purposes.

Prevention of scarring

Methods Used to Prevent the Development of Hypertrophic Scars and/or Keloids in Patients With Expected Uneventful Wound Healing

**Figure 5:**
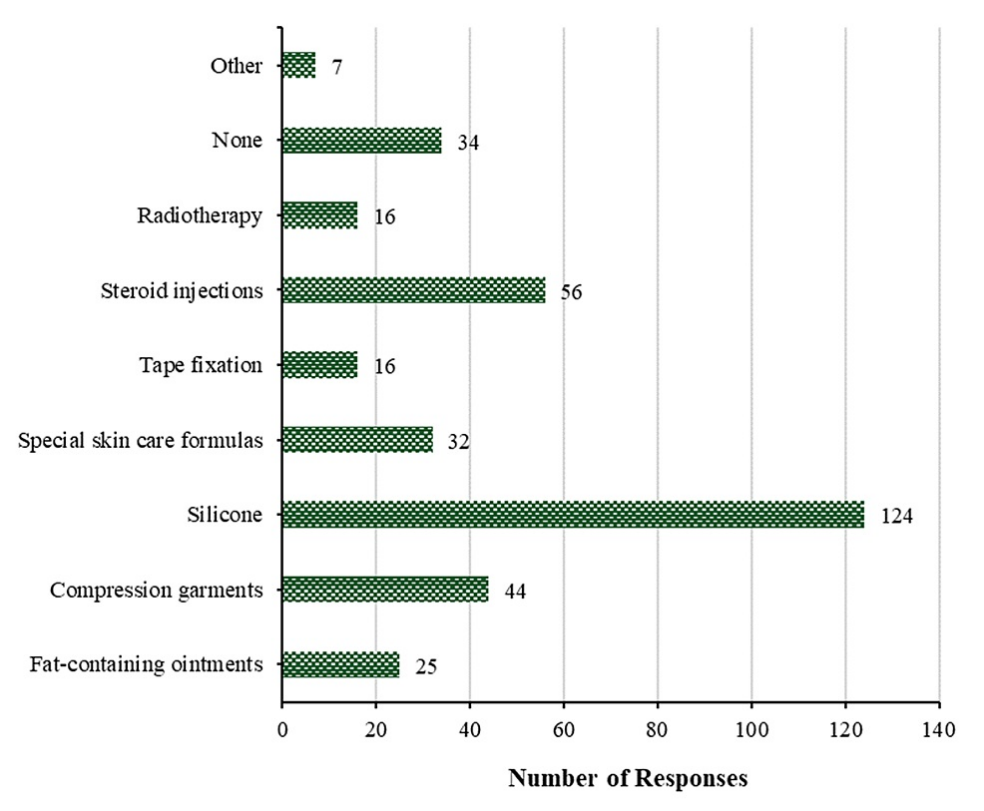
Prevention of scaring. Methods used to prevent the development of hypertrophic scars and/or keloids in patients with expected uneventful wound healing

To mitigate the occurrence of hypertrophic scars and keloids in patients who are anticipated to experience uneventful wound healing, silicone is utilized by over half of the participants (124 (52.3%)). Steroid injections (56 (23.6%)) are followed by compression garments (44 (18.6%)), special skin care formulations (32 (13.5%)), and fat-containing ointments (25 (10.5%)), then tape fixation and radiotherapy with 16 (6.8%) for each, while only 7 (3.0%) participants use other methods. These percentages can be seen in Figure 5. Conversely, a subset of participants (34 (14.3%)) did not implement any preventive measures against the formation of hypertrophic scars and keloids in patients whose wound recovery was anticipated to be uneventful.

Methods Used to Prevent the Development of Hypertrophic Scars and/or Keloids in Patients With an Increased Risk for Developing Hypertrophic Scars

**Figure 6:**
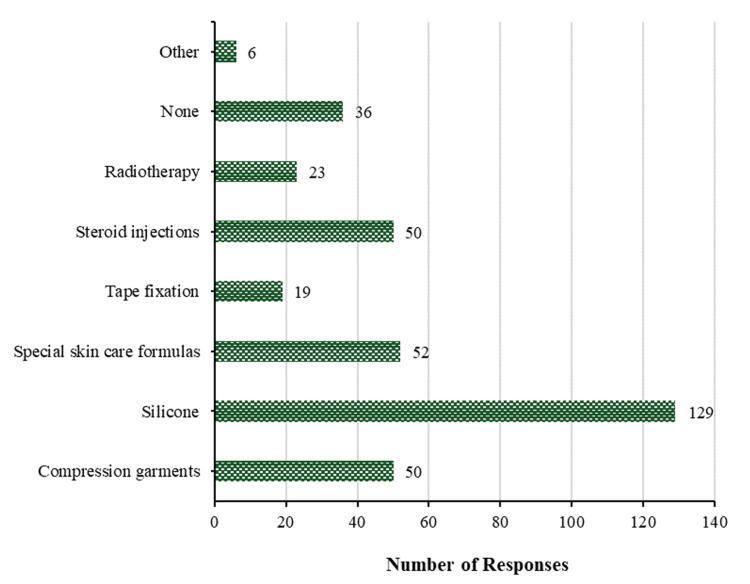
Prevention of scaring. Methods used to prevent the development of hypertrophic scars and/or keloids in patients with an increased risk of developing hypertrophic scars

In patients at heightened risk for developing hypertrophic scars, silicone is the method most frequently used to prevent the formation of keloids and hypertrophic scars (129 (54.4%)). Special skin care formulations follow closely behind at 52 (21.9%), followed by compression garments and steroid injections at 50 (21.1%) each, radiotherapy at 23 (9.7%), tape fixation at 19 (8.0%), and finally alternative methods at only 6 (2.5%). All methods can be seen in Figure 6. Conversely, 36 (15.2%) participants did not utilize any preventative measures against hypertrophic scars and keloids in patients who have an elevated risk of developing such scars.

Methods Used to Prevent the Development of Hypertrophic Scars and/or Keloids in Patients With a Previous History of Keloids

**Figure 7:**
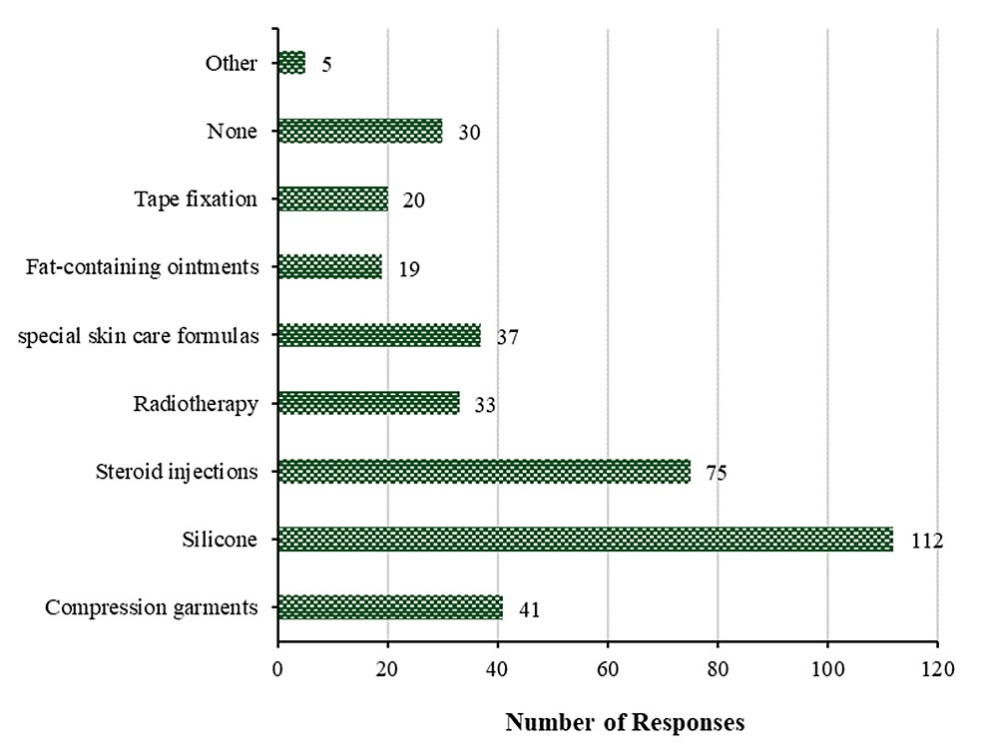
Prevention of scaring. Methods used to prevent the development of hypertrophic scars and/or keloids in patients with a previous history of keloids

Figure 7 reveals that among participants with a prior keloid disease history, the utilization of silicone is the most prevalent method (112 (47.3%)) employed to prevent the formation of hypertrophic scars and keloids. Steroid injections are the second most frequently utilized method (75 (31.6%)), followed by compression garments (41 (17.3%)), special skin care formulas (37 (15.6%)), and radiotherapy (33 (13.9%)). On the other hand, the least frequent methods are tape fixation and fat-containing ointments, with 20 (8.4%) and 19 (8.0%), respectively, while only 5 (2.1%) participants use other methods. However, 30 (12.7%) participants did not take any actions to prevent the development of hypertrophic scars and/or keloids.

Treatment of scarring

Treatment of Hypertrophic Scars

**Figure 8:**
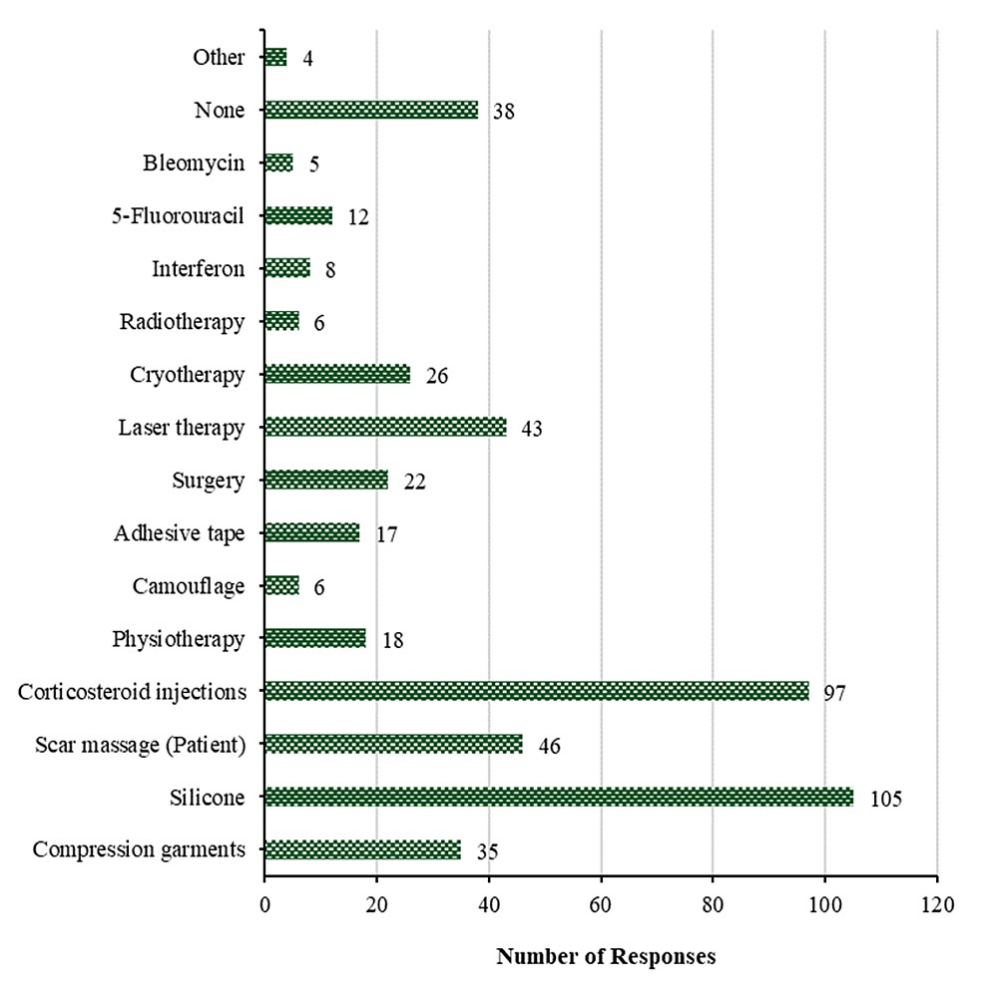
Treatment of hypertrophic scars <18 months

Treatment of hypertrophic scars for less than 18 months: Figure 8 discusses the treatment of hypertrophic scars that are less than 18 months in duration. Silicone is the most frequently used method to treat hypertrophic scars in patients <18 months after initial presentation (105 (44.3%)), followed by corticosteroid injections (97 (40.9%)), scar massage (patient) (46 (19.4%)), laser therapy (43 (18.1%)), compression garments (35 (14.8%)), and cryotherapy (26 (11.0%)). Other modalities chosen by physicians were surgery, physiotherapy, adhesive tape, 5-fluorouracil, and interferon, represented by 22 (9.3%), 18 (7.6%), 17 (7.2%), 12 (5.1%), and 8 (3.4%), respectively. The least common treatments chosen among the participants were camouflage and radiotherapy, with 6 (2.5%) for each; bleomycin was used by 5 (2.1%); and only 4 (1.7%) used other non-specified methods. On the other hand, for patients who report less than 18 months following the initial presentation, 38 (16.0%) participants do nothing to treat hypertrophic scars.

**Figure 9:**
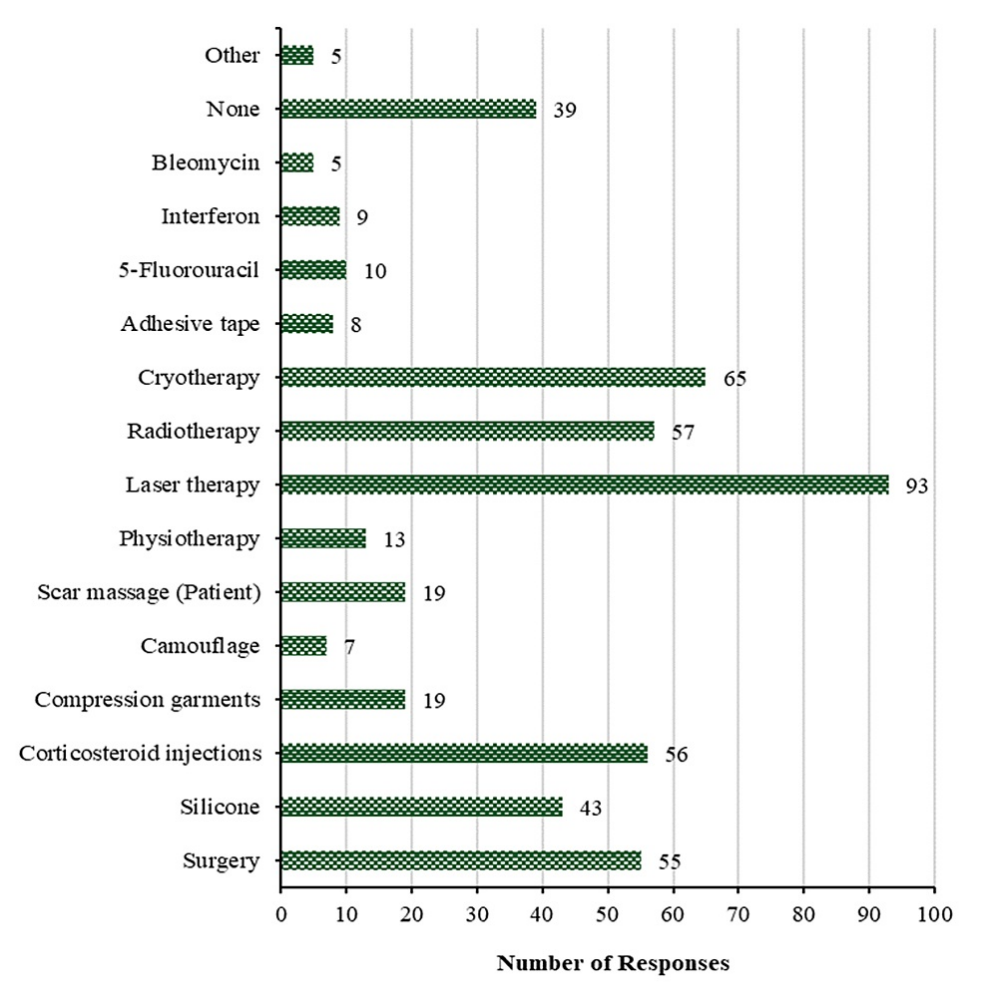
Treatment of hypertrophic scars >18 months

Treatment of hypertrophic scars for more than 18 months: Figure 9 examines the most commonly used methods by participants to treat hypertrophic scars in patients diagnosed for more than 18 months at the time of initial presentation. Laser therapy is the most frequently used method (93 (39.2%)), followed by cryotherapy (65 (27.4%)), radiotherapy (57 (24.1%)), corticosteroid injections (56 (23.6%)), surgery (55 (23.2%)), silicone (43 (18.1%)), then compression garments and scar massage (patient) with 19 (8.0%) for each, while the lowest methods used were namely physiotherapy (13 (5.5%)), then 5-fluorouracil (10 (4.2%)), then interferon (9 (3.8%)), then adhesive tape (8 (3.4%)), then camouflage (7 (3.0%)), then bleomycin and other methods with 5 (2.1%) for each. On the other hand, 39 (16.5%) participants used nothing to treat hypertrophic scars for patients >18 months after initial presentation.

Treatment of keloids

How Do You Treat Keloids? Do You Operate on Them? How Do You Operate on Them?

**Figure 10:**
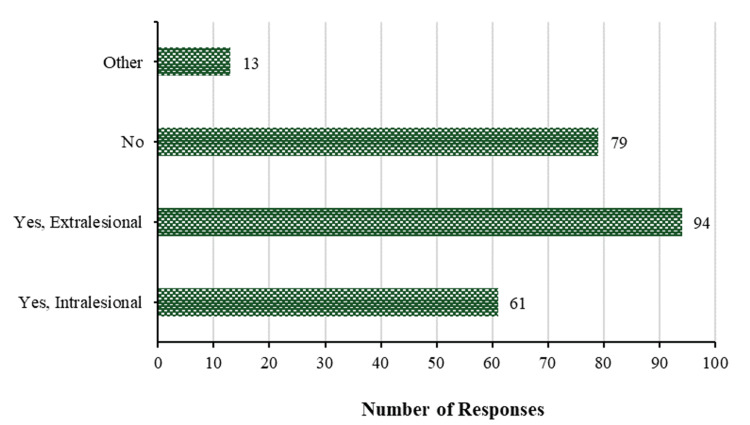
Treatment of keloids. How do you treat keloids? Do you operate on them? How do you operate on them?

Figure 10 highlights the relation to the treatment, operation, and method of operation on keloids, the results indicate that 94 (39.7%) reported performing extralesional procedures to treat keloids, while 61 (25.7%) stated that intralesional procedures were utilized to treat keloids. However, a minority of participants (13 (5.5%)) have alternative methods for treating keloids, with 79 (33.3%) participants not employing any treatment.

How Do You Treat Them Before Surgery?

**Figure 11:**
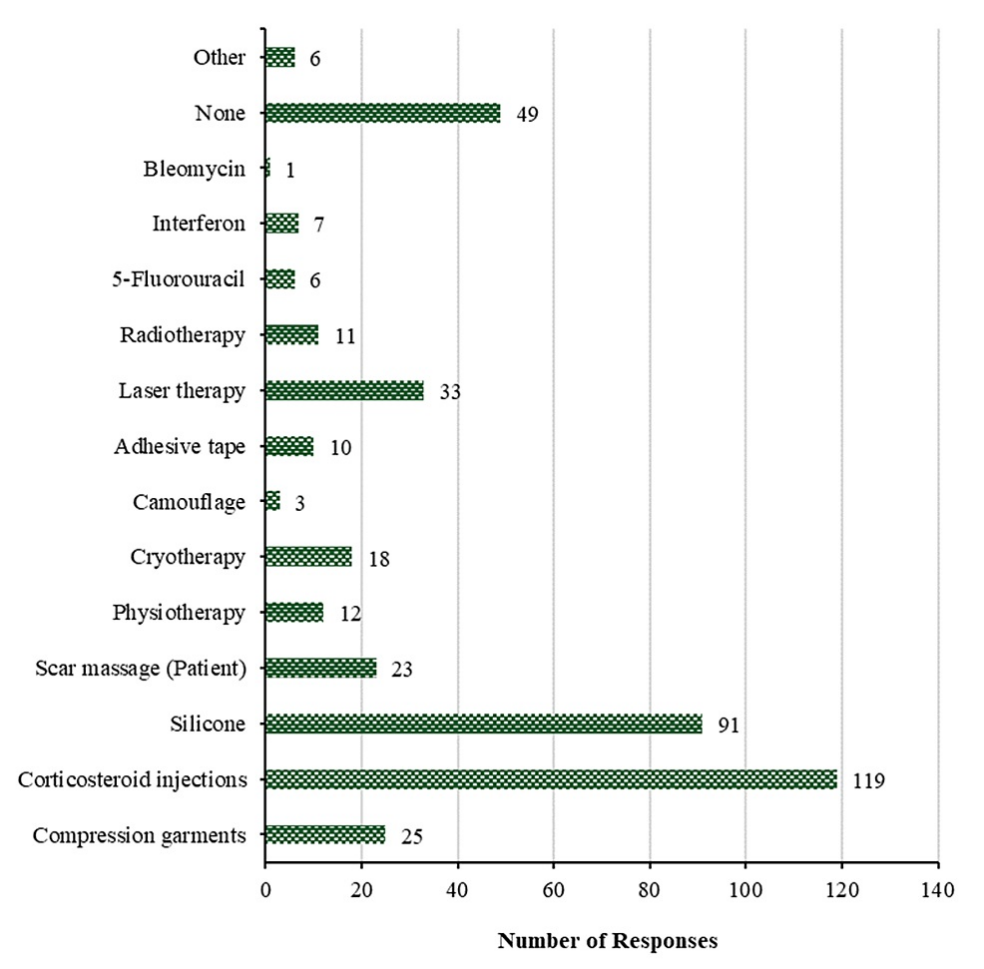
Treatment of keloids. How do you treat them before surgery?

In terms of keloidal pre-operative treatment, Figure 11 reveals that a majority of participants (119 (50.2%)) employ corticosteroid injections. This is followed by silicone (91 (38.4%)), laser therapy (33 (13.9%)), compression garments (25 (10.5%)), and scar massage (patient) (23 (9.7%)). Conversely, cryotherapy is the least utilized method (18 (7.6%)), with physiotherapy (12 (5.1%)) and radiotherapy (11 (4.6%)), adhesive tape (10 (4.2%)), interferon (7 (3.0%)), then 5-fluorouracil and other methods with 6 (2.5%) for each, then camouflage with 3 (1.3%) and bleomycin with 1 (0.4%). The remaining participants (49 (20.7%)), however, do not apply any treatment to keloids prior to surgery.

How Do You Treat Them After Surgery?

**Figure 12:**
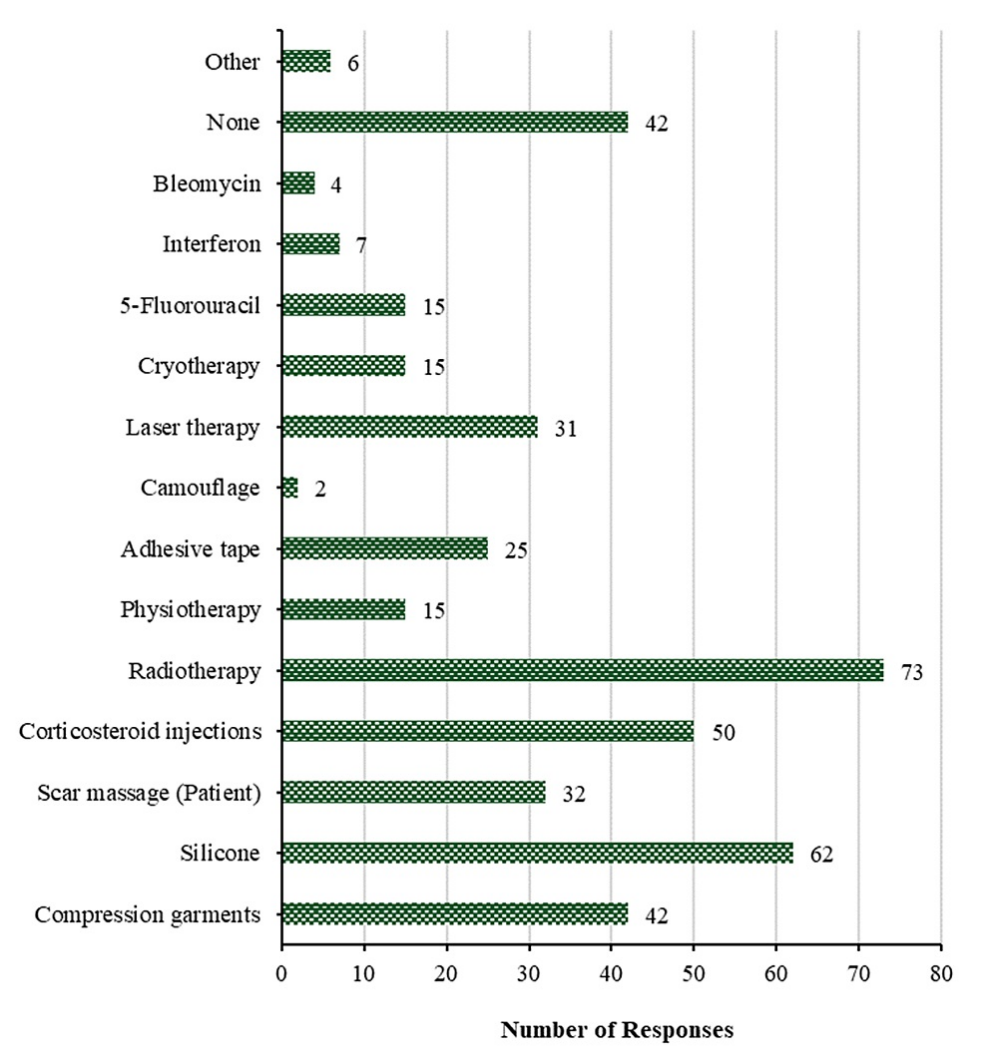
Treatment of keloids. How do you treat them after surgery?

Regarding the management of keloids after surgery, Figure 12 demonstrates the most common methods namely radiotherapy (73 (30.8%)), followed by silicone (62 (26.2%)), then corticosteroid injections (50 (21.1%)), then compression garments (42 (17.7%)), then scar massage (patient) (32 (13.5%)), then laser therapy (31 (13.1%)), then adhesive tape (25 (10.5%)). The least frequent methods for treating keloids are physiotherapy, cryotherapy, and 5-fluorouracil with 15 (6.3%) for each, interferon (7 (3.0%)), other methods (6 (2.5%)), bleomycin (4 (1.7%)), and camouflage (2 (0.8%)). On the other hand, there are 42 (17.7%) participants doing nothing to treat keloids after surgery.

Non-surgical Treatment of Keloids

**Figure 13:**
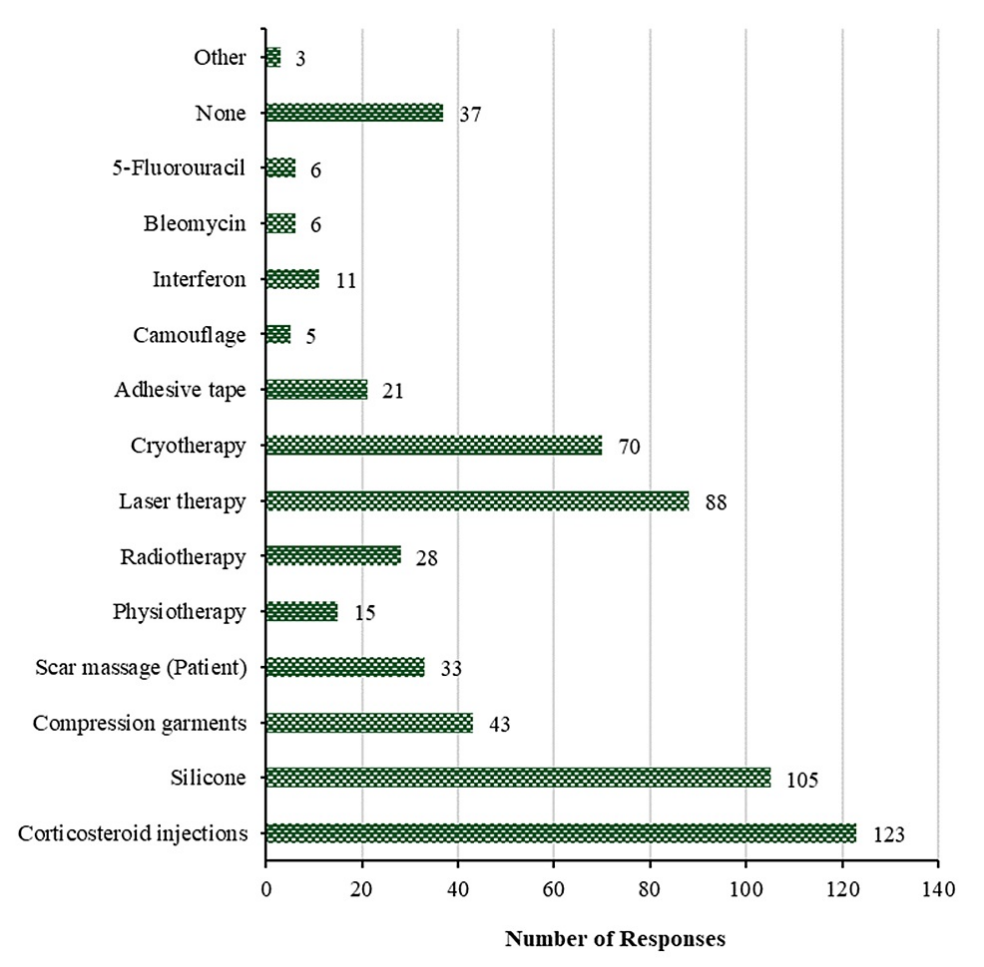
Treatment of keloids. Non-surgical treatment of keloids

In terms of non-surgical treatment of keloids, it is found that the most common methods are corticosteroid injections (123 (51.9%)), followed by silicone (105 (44.3%)), then laser therapy (88 (37.1%)), then cryotherapy (70 (29.5%)), then compression garments (43 (18.1%)), then scar massage (patient) (33 (13.9%)), then radiotherapy (28 (11.8%)). The least used methods of non-surgical treatment of keloids are adhesive tape (21 (8.9%)), followed by physiotherapy (15 (6.3%)), then interferon (11 (4.6%)), then bleomycin and 5-fluorouracil with 6 (2.5%) for each, then camouflage (5 (2.1%)), and the other methods (3 (1.3%)). However, 37 (15.6%) participants haven't used the non-surgical treatment of keloids. The summary of responses is shown in Figure 13.

## Discussion

Evaluation methods

The study offers insightful information about methods for evaluating scars that are applied in research and clinical settings. Subjectively the frequency with which both patient and observer scar assessments are conducted emphasizes the need to include patient viewpoints in scar evaluation and the necessity of taking a comprehensive approach to comprehending scar outcomes [16,17]. Furthermore, the utilization of corresponding scars and photograph assessments highlights the significance of visual aids in subjective evaluations, enabling more precise and thorough evaluations [18].

The existence of diverse subjective assessment techniques, however, raises the possibility of difficulties in harmonizing scar evaluation protocols between healthcare settings [19,20]. Although some methods, such as the Vancouver Scar Scale, are often used, a significant portion of participants favor customized or alternative approaches. This emphasizes how crucial it is for scar assessment techniques to be adaptable to account for a range of patient variables and clinician preferences.

The Modified Vancouver Scar Scale is preferred in research settings, which suggests that it is well-accepted and useful in scientific studies [21]. However, given the variety of study methodologies and the sizeable fraction of participants who do not employ any standardized technique, consensus standards are necessary to ensure comparability and consistency in scar evaluation for scientific reasons. Creating uniform rules could improve the validity and reliability of scar assessment in research, leading to more substantial evidence generation and improving our knowledge of scar care techniques.

Regarding the objective methods used to evaluate scars, two-dimensional photography has become the standard objective method in clinical practice since it provides a visual record of scar growth and makes longitudinal examinations easier [22]. The substantial percentage of participants who did not use any objective method, however, points to possible weaknesses in clinical practice and calls for a more thorough integration of objective assessment procedures.

On the other hand, biomechanical characteristics became the most common objective method for scar assessment in research settings, emphasizing the importance of quantitative metrics in scientific inquiry. However, the underuse of some objective techniques like colorimetry and computer-aided design indicates that more research is necessary to determine how well they work as scar assessment tools. This emphasizes how crucial it is to investigate a range of objective methods to guarantee thorough and precise scar assessment in both clinical and research settings.

Preventive measures

The study delves into the prevention and management of keloids and hypertrophic scars across various patient populations, highlighting the widespread use of silicone as the primary preventive measure due to its proven efficacy and safety [23]. Additionally, therapies such as steroid injections, compression garments, and specialized skincare products are commonly employed, reflecting a multifaceted approach aimed at targeting different aspects of scar pathophysiology [24].

While silicone remains the preferred option across all risk groups, individuals at higher risk for hypertrophic scarring may benefit from additional therapies like radiation and tape fixation, demonstrating the importance of tailored treatment strategies based on individual risk assessment.

For individuals with a history of keloids, silicone and steroid injections are frequently utilized to regulate abnormal wound healing responses and prevent recurrence. However, the study reveals a significant portion of participants who do not undertake any preventive measures despite their keloid history, indicating potential gaps in clinical practice and the need for enhanced education among healthcare providers.

Treatment approaches

The treatment of hypertrophic scars within and beyond 18 months of onset showcases a shift in therapeutic strategies. Corticosteroid injections and silicone are primary interventions within the initial 18-month period, aiming to mitigate inflammation and encourage scar maturation. Additionally, compression garments, laser therapy, and scar massage highlight the comprehensive approach adopted by healthcare professionals to address hypertrophic scarring.

However, beyond the 18-month mark, laser therapy becomes the predominant intervention, indicating a transition toward treatments focused on tissue restructuring and scar remodeling. Cryotherapy and radiation therapy also gain prominence, providing additional options for scar reduction and symptom alleviation [25].

Despite this, a study done in Korea specified the approach to scarring by early intervention as a key to controlling hyperplastic response. Hypertrophic scars that do not improve by six months should be managed aggressively with intralesional steroid injections and alternative modalities. Silicone gel sheets proved to be effective in limiting the hypertrophic growth of scars and are believed to decrease scar size. Pressure therapy can prevent some scar elevation and should be started soon after clinical wound healing in severe cases. Additionally, various forms of lasers, including fractional laser, pulsed dye laser, and intense pulsed light, have been reported to improve scar appearance. Authors have found that radiation therapy was associated with an average decrease of 55% in keloid scars at 30 months of follow-up. Alternative approaches described in keloid literature include chemotherapy agents, 5-fluorouracil, and bleomycin [26].

Notably, a proportion of individuals opt not to pursue treatment for hypertrophic scars, especially beyond 18 months, underscoring potential variations in clinical practice and highlighting the complexities involved in managing adult hypertrophic scars. This suggests a need for further research to understand the rationale behind non-interventionist approaches and to optimize therapeutic strategies for this patient population.

Case-specific conservative treatments should be implemented, including gel sheeting, taping fixation, compression therapy, external and internal agents, and makeup (camouflage) therapy.

Keloid treatment encompasses various surgical and non-surgical interventions tailored to individual patient needs and preferences. Surgical options, such as intralesional and extralesional techniques, offer precise and complete removal of keloid tissue, with patient preference and keloid location influencing the choice between these approaches. However, a notable percentage of participants opt against surgical intervention, indicating a preference for non-invasive or alternative methods [27].

An algorithm for treating keloid/hypertrophic scars was developed in a Japanese study. It has been stated that the treatment approaches for keloids are contingent upon their size, number, and scar characteristics. In the case of small and single keloids, nonsurgical monotherapy including cryotherapy, laser, corticosteroid injections, or 5-fluorouracil may be utilized in addition to radiation or corticosteroid injections during surgery. Nevertheless, keloids that are sizable and numerous are challenging to treat radically and can only be managed with multimodal therapies that aim to alleviate symptoms. [10]. On the other hand, an investigation in Texas underscored the lack of evidence supporting the superiority of any particular product or approach. Moreover, it revealed that physicians tended to tailor treatment plans around non-surgical multimodal therapies with the aim of optimizing the aesthetic appeal of scars. Given the 12- to 18-month maturation period for scars, surgical scar revision should be deliberated upon only after more conservative treatment options have been exhausted. Revision surgery may be employed to rectify the visibility of an undesired scar and restore its aesthetic appeal [28].

Pre-operative strategies focus on reducing keloid recurrence and optimizing surgical outcomes, with silicone therapy and corticosteroid injections being the most commonly utilized options due to their efficacy in reducing inflammation and keloid growth. Additionally, scar massage, laser therapy, and compression garments complement these interventions, reflecting a comprehensive approach to preparing keloids for surgery.

Post-operative management aims to promote wound healing and minimize keloid recurrence, primarily through radiotherapy and silicone therapy. Scar massage, compression garments, and corticosteroid injections continue to be employed post-operatively to reduce inflammation and encourage collagen remodeling. However, a significant proportion of individuals do not receive post-operative care, highlighting potential variations in clinical practice and the need for further research to elucidate reasons behind non-interventionist approaches.

Non-surgical treatments offer alternative options for managing keloids, especially when surgery may not be suitable. Corticosteroid injections, silicone therapy, and laser therapy play pivotal roles in reducing inflammation and remodeling keloid tissue. Cryotherapy, compression garments, and scar massage further expand the range of non-surgical interventions available, providing patients with diverse treatment modalities [29].

Limitations and recommendations

While this study offers insightful information, there are some important limitations to be aware of. There are limitations in establishing causality or tracking changes over time with the cross-sectional approach. In addition, response bias could be introduced by the study's dependence on self-reported data.

Future studies could use longitudinal designs to evaluate how practice patterns have changed over time to overcome these constraints. Furthermore, qualitative research examining medical doctors' opinions and experiences with scar management may offer a more profound understanding of the processes involved in decision-making and the obstacles to the best possible care.

## Conclusions

Despite the advancements in scar management, there remains a need for standardized approaches and greater adherence to evidence-based guidelines. By bridging the gap between research and clinical practice, healthcare professionals can enhance patient outcomes and contribute to the continuous improvement of scar management strategies. Further collaboration between researchers, clinicians, and policymakers is essential for advancing the field of scar management and improving the quality of care for individuals affected by scars.

## Appendices

**Table 3:** Checklist for Reporting Results of Internet E-Surveys (CHERRIES) Reference: [15]

Checklist Item	Explanation
Describe survey design	Describe target population, sample frame. Is the sample a convenience sample? (In “open” surveys this is most likely.)
IRB approval	Mention whether the study has been approved by an IRB.
Informed consent	Describe the informed consent process. Where were the participants told the length of time of the survey, which data were stored and where and for how long, who the investigator was, and the purpose of the study?
Data protection	If any personal information was collected or stored, describe what mechanisms were used to protect unauthorized access.
Development and testing	State how the survey was developed, including whether the usability and technical functionality of the electronic questionnaire had been tested before fielding the questionnaire.
Open survey versus closed survey	An “open survey” is a survey open for each visitor of a site, while a closed survey is only open to a sample which the investigator knows (password-protected survey).
Contact mode	Indicate whether or not the initial contact with the potential participants was made on the Internet. (Investigators may also send out questionnaires by mail and allow for Web-based data entry.)
Advertising the survey	How/where was the survey announced or advertised? Some examples are offline media (newspapers), or online (mailing lists – If yes, which ones?) or banner ads (Where were these banner ads posted and what did they look like?). It is important to know the wording of the announcement as it will heavily influence who chooses to participate. Ideally the survey announcement should be published as an appendix.
Web/e-mail	State the type of e-survey (eg, one posted on a Web site, or one sent out through e-mail). If it is an e-mail survey, were the responses entered manually into a database, or was there an automatic method for capturing responses?
Context	Describe the Web site (for mailing list/newsgroup) in which the survey was posted. What is the Web site about, who is visiting it, what are visitors normally looking for? Discuss to what degree the content of the Web site could pre-select the sample or influence the results. For example, a survey about vaccination on an anti-immunization Web site will have different results from a Web survey conducted on a government Web site
Mandatory/voluntary	Was it a mandatory survey to be filled in by every visitor who wanted to enter the Web site, or was it a voluntary survey?
Incentives	Were any incentives offered (eg, monetary, prizes, or non-monetary incentives such as an offer to provide the survey results)?
Time/date	In what timeframe were the data collected?
Randomization of items or questionnaires	To prevent biases items can be randomized or alternated.
Adaptive questioning	Use adaptive questioning (certain items, or only conditionally displayed based on responses to other items) to reduce number and complexity of the questions.
Number of items	What was the number of questionnaire items per page? The number of items is an important factor for the completion rate.
Number of screens (pages)	Over how many pages was the questionnaire distributed? The number of items is an important factor for the completion rate.
Completeness check	It is technically possible to do consistency or completeness checks before the questionnaire is submitted. Was this done, and if “yes”, how (usually JAVAScript)? An alternative is to check for completeness after the questionnaire has been submitted (and highlight mandatory items). If this has been done, it should be reported. All items should provide a non-response option such as “not applicable” or “rather not say”, and selection of one response option should be enforced.
Review step	State whether respondents were able to review and change their answers (eg, through a Back button or a Review step which displays a summary of the responses and asks the respondents if they are correct).
Unique site visitor	If you provide view rates or participation rates, you need to define how you determined a unique visitor. There are different techniques available, based on IP addresses or cookies or both.
View rate (ratio of unique survey visitors/unique site visitors)	Requires counting unique visitors to the first page of the survey, divided by the number of unique site visitors (not page views!). It is not unusual to have view rates of less than 0.1 % if the survey is voluntary.
Participation rate (ratio of unique visitors who agreed to participate/unique first survey page visitors)	Count the unique number of people who filled in the first survey page (or agreed to participate, for example by checking a checkbox), divided by visitors who visit the first page of the survey (or the informed consents page, if present). This can also be called “recruitment” rate.
Completion rate (ratio of users who finished the survey/users who agreed to participate)	The number of people submitting the last questionnaire page, divided by the number of people who agreed to participate (or submitted the first survey page). This is only relevant if there is a separate “informed consent” page or if the survey goes over several pages. This is a measure for attrition. Note that “completion” can involve leaving questionnaire items blank. This is not a measure for how completely questionnaires were filled in. (If you need a measure for this, use the word “completeness rate”.)
Cookies used	Indicate whether cookies were used to assign a unique user identifier to each client computer. If so, mention the page on which the cookie was set and read, and how long the cookie was valid. Were duplicate entries avoided by preventing users access to the survey twice; or were duplicate database entries having the same user ID eliminated before analysis? In the latter case, which entries were kept for analysis (eg, the first entry or the most recent)?
IP check	Indicate whether the IP address of the client computer was used to identify potential duplicate entries from the same user. If so, mention the period of time for which no two entries from the same IP address were allowed (eg, 24 hours). Were duplicate entries avoided by preventing users with the same IP address access to the survey twice; or were duplicate database entries having the same IP address within a given period of time eliminated before analysis? If the latter, which entries were kept for analysis (eg, the first entry or the most recent)?
Log file analysis	Indicate whether other techniques to analyze the log file for identification of multiple entries were used. If so, please describe.
Registration	In “closed” (non-open) surveys, users need to login first and it is easier to prevent duplicate entries from the same user. Describe how this was done. For example, was the survey never displayed a second time once the user had filled it in, or was the username stored together with the survey results and later eliminated? If the latter, which entries were kept for analysis (eg, the first entry or the most recent)?
Handling of incomplete questionnaires	Were only completed questionnaires analyzed? Were questionnaires which terminated early (where, for example, users did not go through all questionnaire pages) also analyzed?
Questionnaires submitted with an atypical timestamp	Some investigators may measure the time people needed to fill in a questionnaire and exclude questionnaires that were submitted too soon. Specify the timeframe that was used as a cut-off point, and describe how this point was determined.
Statistical correction	Indicate whether any methods such as weighting of items or propensity scores have been used to adjust for the non-representative sample; if so, please describe the methods.
